# Multi-epitope mRNA vaccine and protein vaccine protect mice against *Toxoplasma gondii*

**DOI:** 10.1186/s13071-026-07447-5

**Published:** 2026-06-05

**Authors:** Yulin Cui, Hongnan Qu, Chunxue Zhou, Ziyue Liang, Bing Han, Huaiyu Zhou, Hua Cong

**Affiliations:** Department of Pathogenic Biology, School of Basic Medical Sciences, Cheeloo College of Medicine, Shandong University, Jinan, 250012 Shandong People’s Republic of China

**Keywords:** *Toxoplasma gondii*, Multi-epitope vaccine, mRNA vaccine, Lipid nanoparticle (LNP), Recombinant protein vaccine, Protective immunity

## Abstract

**Background:**

*Toxoplasma gondii*, an obligate intracellular protozoan parasite, infects almost one-third of the world’s population and all warm-blooded animals, posing a substantial threat to public health. Accordingly, the development of effective vaccines against *T. gondii* has become an urgent priority. In this study, we constructed a multi-epitope chimeric antigen T-SGR targeting three key protective antigens of *T. gondii* (SAG1, GRA7, and ROP16), and developed both a messenger RNA (mRNA) lipid nanoparticle (LNP) vaccine and a recombinant protein vaccine based on T-SGR. The immunogenicity and protective effects were further evaluated in C57BL/6 mice.

**Methods:**

The T-SGR mRNA–LNP vaccine was prepared via in vitro transcription followed by LNP encapsulation, while the T-SGR protein vaccine was obtained via prokaryotic expression and purification. Mice were administered a two-dose immunization regimen. Serum levels of specific IgG, IgG1, and IgG2a antibodies and cytokine levels were measured by enzyme-linked immunosorbent assay (ELISA). T lymphocyte subsets and lymphocyte proliferation were assessed by flow cytometry and Cell Counting Kit-8 (CCK-8) assay. Protective efficacy was evaluated by monitoring survival rates after challenge with highly virulent *T. gondii* RH strain tachyzoites and moderately virulent ME49 strain tachyzoites.

**Results:**

Both T-SGR mRNA and protein vaccines induced robust humoral and cellular immune responses in mice. Notably, the IgG antibody titer induced by the mRNA–LNP vaccine was significantly higher than that of the protein vaccine (*P* < 0.05). Both vaccines drove a Th1-biased immune response, as evidenced by markedly higher IgG2a levels relative to IgG1. Compared with the phosphate-buffered saline (PBS) control group, both vaccine groups significantly promoted splenocyte proliferation (*P* < 0.05). The mRNA vaccine induced significantly higher secretion of IFN-γ, IL-10, IL-12, and IL-2 than the protein vaccine. Both vaccines conferred significant protection against *T. gondii* infection and prolonged mouse survival. Strikingly, the T-SGR mRNA–LNP vaccine provided 100% protection against the *T. gondii* ME49 strain, outperforming the recombinant protein vaccine.

**Conclusions:**

We successfully developed a multi-epitope T-SGR mRNA–LNP vaccine and a recombinant protein vaccine against *T. gondii*. The T-SGR mRNA–LNP vaccine elicited stronger humoral and cellular immune responses and conferred superior protective efficacy, representing a promising candidate vaccine against toxoplasmosis.

**Supplementary Information:**

The online version contains supplementary material available at 10.1186/s13071-026-07447-5.

## Background

Toxoplasmosis, a globally widespread parasitic disease caused by the obligate intracellular protozoan *T. gondii*, affects approximately one-third of the world’s population [1, 2]. In China, the estimated seroprevalence of human infection ranges from 5% to 10% [3, 4].

Although most infections in immunocompetent individuals are asymptomatic or mild, *T. gondii* can cause severe and even life-threatening diseases in immunocompromised patients, such as those with acquired immune deficiency syndrome (AIDS), organ transplantation, or long-term immunosuppressive therapy [5, 6]. Moreover, primary infection during pregnancy may lead to transplacental transmission, resulting in fetal malformation, stillbirth, or neurological sequelae [7, 8]. Therefore, developing safe, effective, and broadly protective vaccines against *T. gondii* is of great significance for public health. Over recent decades, research on *T. gondii* vaccines has evolved from traditional inactivated vaccines, live attenuated vaccines, and crude antigen vaccines to genetically engineered vaccines, including subunit vaccines, DNA vaccines, viral vector vaccines, and messenger RNA (mRNA) vaccines [9, 10]. However, traditional vaccines have obvious limitations: Live attenuated vaccines carry potential risks of virulence reversion; inactivated and protein vaccines often induce insufficient cellular immunity; and single-antigen vaccines fail to provide broad-spectrum protection against different strains and life stages of *T. gondii* [11, 12]. With the development of immune informatics and epitope prediction, multi-epitope vaccine design has become a rational and promising strategy. By integrating immunodominant B cell and T cell epitopes from multiple key antigens, multi-epitope vaccines can broaden immune recognition, enhance protective breadth, and avoid immune interference between antigens [13, 14].

Among numerous *T. gondii* antigens, SAG1, GRA7, and ROP16 have been widely recognized as ideal candidate antigens. SAG1 is a major surface antigen of tachyzoites that mediates host cell adhesion and invasion, with strong immunogenicity [15]. GRA7 is a dense granule protein stably expressed in tachyzoites and bradyzoites, which maintains the parasitophorous vacuole structure and is closely related to parasite survival and virulence [16]. ROP16 is a rhoptry kinase that can be translocated into the host nucleus to regulate immune signaling pathways and promote immune evasion [17]. These three antigens participate in invasion, intracellular survival, and immune escape, respectively, with complementary functions and broad protective potential.

As an emerging vaccine platform, mRNA vaccines have shown unique advantages in prevention of infectious diseases. mRNA vaccines can be rapidly designed and produced without complex protein expression and purification [18]. They are translated endogenously in host cells, inducing both humoral and cellular immune responses similar to natural infection [19, 20]. In addition, lipid nanoparticle (LNP) delivery systems significantly improve the stability, translation efficiency, and immunogenicity of mRNA [21, 22]. At present, systematic comparisons between mRNA vaccines and recombinant protein vaccines based on the same multi-epitope antigen against *T. gondii* remain limited.

In this study, we designed and constructed a multi-epitope chimeric antigen T-SGR by fusing key immunodominant fragments of SAG1, GRA7, and ROP16. We further prepared a T-SGR mRNA–LNP vaccine and a recombinant protein vaccine, and systematically compared their humoral immunity, cellular immunity, and protective efficacy in C57BL/6 mice. This study aims to identify an optimal vaccine platform against *T. gondii* and provide an experimental basis for the development of next-generation toxoplasmosis vaccines.

## Methods

### Construction of the *T. gondii* T-SGR multi-epitope gene

Gene sequences of *T. gondii* *SAG1*, *GRA7*, and *ROP16* were retrieved from the National Center for Biotechnology Information (NCBI) GenBank database. Immunodominant epitope regions were screened using DNASIS version 2.6 and the IEDB online tool, based on B cell epitope antigenicity index (> 1.0) and major histocompatibility complex (MHC) binding affinity of CTL/Th epitopes (eluted ligand rank score < 2%). The selected fragments were SAG1 (160–260 aa), GRA7 (140–236 aa), and ROP16 (230–380 aa). These fragments were linked in tandem using the flexible SAPGTP linker to construct the chimeric multi-epitope gene T-SGR (Supplementary Fig. S1A). Physicochemical properties of T-SGR were analyzed using ExPASy ProtParam. Three-dimensional structure prediction was performed using AlphaFold3 to verify the folding state and epitope accessibility (Supplementary Fig. S1B).

### Animals and parasites

Specific pathogen-free (SPF) female C57BL/6 mice (6 weeks old) were purchased from Beijing Charles River Laboratories (certification no. SYXK 2020-0012) and housed under standardized conditions (22 °C, 12 h light/dark cycle). All animal procedures were approved by the Institutional Animal Care and Use Committee of Shandong University (no. 20230003) and performed in accordance with national guidelines GB/T 35892-2018.

*T. gondii* RH strain (type I, highly virulent) and ME49 strain (type II, moderately virulent) were propagated in human foreskin fibroblast (HFF) cells cultured in Dulbecco’s modified Eagle medium (DMEM; cat. no. 11995500B, Gibco, USA) supplemented with 10% fetal bovine serum (FBS; cat. no. CF-01P-02; Cell Box, China).

### Expression and purification of T-SGR recombinant protein

The *T-SGR* gene was cloned into the pET-28a vector to generate the recombinant plasmid pET-28a-His-T-SGR. The plasmid was transformed into *Escherichia coli* BL21 competent cells. Positive clones were cultured in 2× yeast extract–tryptone (YT) medium containing 50 μg/mL kanamycin (cat. no. IK00303; Solarbio, Beijing, China) at 37 °C until optical density at 600 nm (OD_600_) reached 0.6. Protein expression was induced with 0.25 mM IPTG (cat. no. II01302; Solarbio, Beijing, China) at 37 °C for 4 h [23].

### Immunization of rats with T-SGR recombinant protein

Female Wistar rats were subcutaneously immunized with 100 μg of purified T-SGR protein emulsified in Freund’s complete adjuvant (cat. no. F5881, Sigma-Aldrich, St. Louis, MO, USA). Two booster immunizations were administered at 7-day intervals. Antisera were collected 7 days after the last immunization, and specificity was verified by western blot.

### T-SGR mRNA synthesis

The linearized T-SGR DNA was amplified by polymerase chain reaction (PCR) using pET-28a-His-T-SGR as the template and purified using a gel extraction kit (cat. no. DP214-02, TIANGEN, Beijing, China). In vitro transcription was performed using a T7 promoter-based transcription kit (cat. no. 16222ES10, Yeasen, Shanghai, China), with cotranscriptional capping and N^1^-methylpseudouridine modification. A poly(A) tail (150 nucleotides [nt]) was added using poly(A) polymerase (cat. no. 14801ES60, Yeasen, Shanghai, China) [24]. Template DNA was digested with DNase I (cat. no. D8075, Solarbio, Beijing, China) at 37 °C for 15 min. mRNA was purified by LiCl precipitation, dissolved in RNase-free water, and quantified using a NanoDrop spectrophotometer. Integrity was verified by denaturing agarose gel electrophoresis and capillary electrophoresis. The capping and tailing efficiency of mRNA was determined by liquid chromatography–mass spectrometry (LC–MS).

### LNP encapsulation of T-SGR mRNA

A lipid–ethanol solution was prepared by mixing SM102, PEG2000-DMG, DSPC, and cholesterol at a molar ratio of 50:1.5:10:38.5. T-SGR mRNA was diluted in citrate buffer (pH 4.0) and mixed with the lipid solution using a microfluidic device (Fluidiclab) at flow rates of 5 mL/min (ethanol) and 15 mL/min (buffer) to formulate mRNA–LNP. The formulation was diluted, ultrafiltered, and buffer-exchanged into Tris–HCl. RNA concentration and encapsulation efficiency were determined using the Quant-iT RiboGreen RNA Assay Kit (cat. no. R11490, Invitrogen, USA). Particle size and morphology were measured using dynamic light scattering (Zetasizer Pro) and transmission electron microscopy (TEM).

### T-SGR mRNA expressed in vitro

HEK293 cells were seeded in 6-well plates and transfected with 3 μg of T-SGR mRNA using Lipofectamine™ 2000 (cat. no. 12566014, Invitrogen, USA) according to the manufacturer’s instructions. Cell lysates were collected at 12 h and 24 h after transfection, and T-SGR protein expression was analyzed by western blotting.

### Western blotting

Total protein was extracted using RIPA lysis buffer (cat. no. P0038, Beyotime) containing protease inhibitors and quantified with a BCA assay kit (cat. no. P0009, Beyotime). Samples were separated by 10% sodium dodecyl sulfate—polyacrylamide gel electrophoresis (SDS–PAGE) and transferred to PVDF membranes. Membranes were blocked with 5% bovine serum albumin (BSA), incubated overnight at 4 °C with primary antibodies (anti-T-SGR, 1:800; anti-GAPDH, 1:5000, cat. no. AF2823, Beyotime, as internal control), followed by horseradish peroxidase (HRP)-conjugated secondary antibody (cat. no. A0192, Beyotime, 1:5000). Protein bands were visualized using a Tanon-5200 imaging system.

### Indirect immunofluorescence assay

HEK293 cells were seeded in 24-well plates and transfected with 2 μg of T-SGR mRNA using Lipofectamine™ 2000 according to the manufacturer’s instructions. At 12 h after transfection, cells were fixed, permeabilized, and blocked sequentially. Cells were incubated with anti-T-SGR primary antibody overnight at 4 °C, followed by incubation with a fluorescent secondary antibody (cat. no. 33308ES60, Yeasen, Shanghai, China) at room temperature in the dark. Finally, cells were stained with DAPI (cat. no. C1002, Beyotime, Shanghai, China) and observed under a fluorescence microscope.

### Mice immunization and sample collection

A total of 45 6-week-old female C57BL/6 mice were randomly divided into three groups (15 mice per group): T-SGR mRNA group, T-SGR protein group, and phosphate-buffered saline (PBS) control group. Mice were intramuscularly immunized twice at a 2-week interval with 10 μg T-SGR mRNA or protein per dose. Control mice received an equal volume of PBS. The entire immunization experiment was repeated twice to verify the stability of the experimental results. Blood samples were collected from the tail vein before immunization and at 2 and 4 weeks after the last immunization. Sera were separated and stored at −20 °C for antibody assays. Then, 2 weeks after the last immunization, spleens were harvested from three mice per group for lymphocyte proliferation and cytokine detection.

### Detection of specific antibodies by enzyme-linked immunosorbent assay (ELISA)

Serum levels of total IgG, IgG1, and IgG2a were measured using commercial ELISA kits according to the manufacturer’s instructions (IgG: cat. no. KYY-0057M1; IgG1: cat. no. KYY-44268M1; IgG2a: cat. no. KYY-0644M1; Research Cloud Biology, China). Optical density (OD) values were read at 450 nm using a microplate reader. Antibody titers were calculated on the basis of standard curves.

### Preparation of splenocyte from immunized mice

Splenocytes were ground and filtered through a 70-μm cell strainer. Red blood cells were lysed with erythrocyte lysis buffer (cat. no. RT122, TIANGEN, Beijing, China). Splenocytes were resuspended in Roswell Park Memorial Institute (RPMI) 1640 medium (cat. no. C11875500BT, Gibco, USA) supplemented with 10% FBS.

### Lymphocyte proliferation assay

Splenocytes (2 × 10^5^ cells) were seeded in 96-well plates. T-SGR-specific antigen was added to a final concentration of 10 μg/mL to stimulate the cells, followed by incubation for 72 h. Then, 10 μL of Cell Counting Kit-8 (CCK-8) reagent (cat. no. BS350B, Biosharp, Beijing, China) was added, and absorbance at 450 nm was measured after 4 h of incubation. The relative proliferation rate was calculated.

### Cytokines assay

Splenocytes (5 × 10^5^ cells) were seeded in 96-well plates. Cells were stimulated with T-SGR protein at a final concentration of 10 μg/mL. Cell culture supernatants were collected at 24 h (IL-2, IL-4), 72 h (IL-10), and 96 h (IFN-γ, IL-12) post-stimulation. Cytokine concentrations were determined using commercial ELISA kits (IL-2: KYY-0701M1; IL-4: KYY-0165M1; IL-10: KYY-0176M2; IFN-γ: KYY-0182M1; IL-12: KYY-0105M1; Research Cloud Biology, China).

### Flow cytometry analysis

Splenocytes (1 × 10^6^ cells) were seeded in 96-well plates. Cells were stimulated with 10 μg/mL T-SGR protein plus Brefeldin A (BFA; cat. no. 50502ES03, Yeasen, Shanghai, China) for 4 h. After stimulation, anti-mouse CD16/32 antibody (cat. no. 101319, Biolegend, USA) was added to block Fc receptors. APC anti-mouse CD45 antibody (cat. no. 147707), PECy5.5 anti-mouse CD3 antibody (cat. no. 100273), FITC anti-mouse CD4 antibody (cat. no. 100405), and PECy7 anti-mouse CD8 antibody (cat. no. 140405) (all from Biolegend, USA) were then added, followed by incubation at 4 °C in the dark for 30 min. Following cell surface staining, cells were fixed with fixation buffer and washed with permeabilization buffer (cat. no. 424401; Biolegend, USA). PE anti-mouse IFN-γ antibody (cat. no. 505807; Biolegend, USA) was added for intracellular staining. Viable lymphocytes were gated according to their characteristic forward scatter (FSC) and side scatter (SSC) profiles to exclude cellular debris. Doublets were eliminated via gating on FSC-H versus FSC-A and SSC-H versus SSC-A. Fluorescence minus one (FMO) controls were included for each marker to ensure accurate gating. Finally, cell subsets were analyzed using a full-spectrum flow cytometer (Powclin, China) with FlowJo software (version 10.8.1) to determine the percentages of CD4^+^ and CD8^+^ T cells and IFN-γ-producing cells.

### *T. gondii* challenge experiment

Mice were intraperitoneally challenged with 100 *T. gondii* RH tachyzoites or 200 ME49 tachyzoites 4 weeks after the last immunization. This challenge experiment was repeated twice independently, with five mice included in each group. Survival and body weight were monitored daily for 30 days. Mice showing severe clinical signs (≥ 20% body weight loss, lethargy, anorexia, or impaired movement) were humanely euthanized.

### Detection of *T. gondii* burden in brain tissue

Surviving mice were euthanized and brain tissues were collected 45 days after challenge. Genomic DNA was extracted using a DNA extraction kit (cat. no. DP304, TIANGEN, Beijing, China). *T. gondii* burden was quantified by quantitative real-time PCR (qPCR) using a qPCR kit (cat. no. B0610A, TIANGEN, Beijing, China).

### Statistical analysis

All data are presented as mean ± standard error of the mean (SEM) from at least three independent biological experiments. Normality was confirmed by the Shapiro–Wilk test. Comparisons between groups were performed using two-tailed Student’s *t*-test, one-way or two-way analysis of variance (ANOVA) followed by Tukey’s multiple comparisons test, or the log-rank (Mantel–Cox) test for survival curves. *P* < 0.05 was considered statistically significant. All statistical analyses were performed using GraphPad Prism 8.0 (GraphPad Software, San Diego, CA, USA).

## Results

### In vitro expression of* T. gondii* T-SGR

**Fig. 1:**
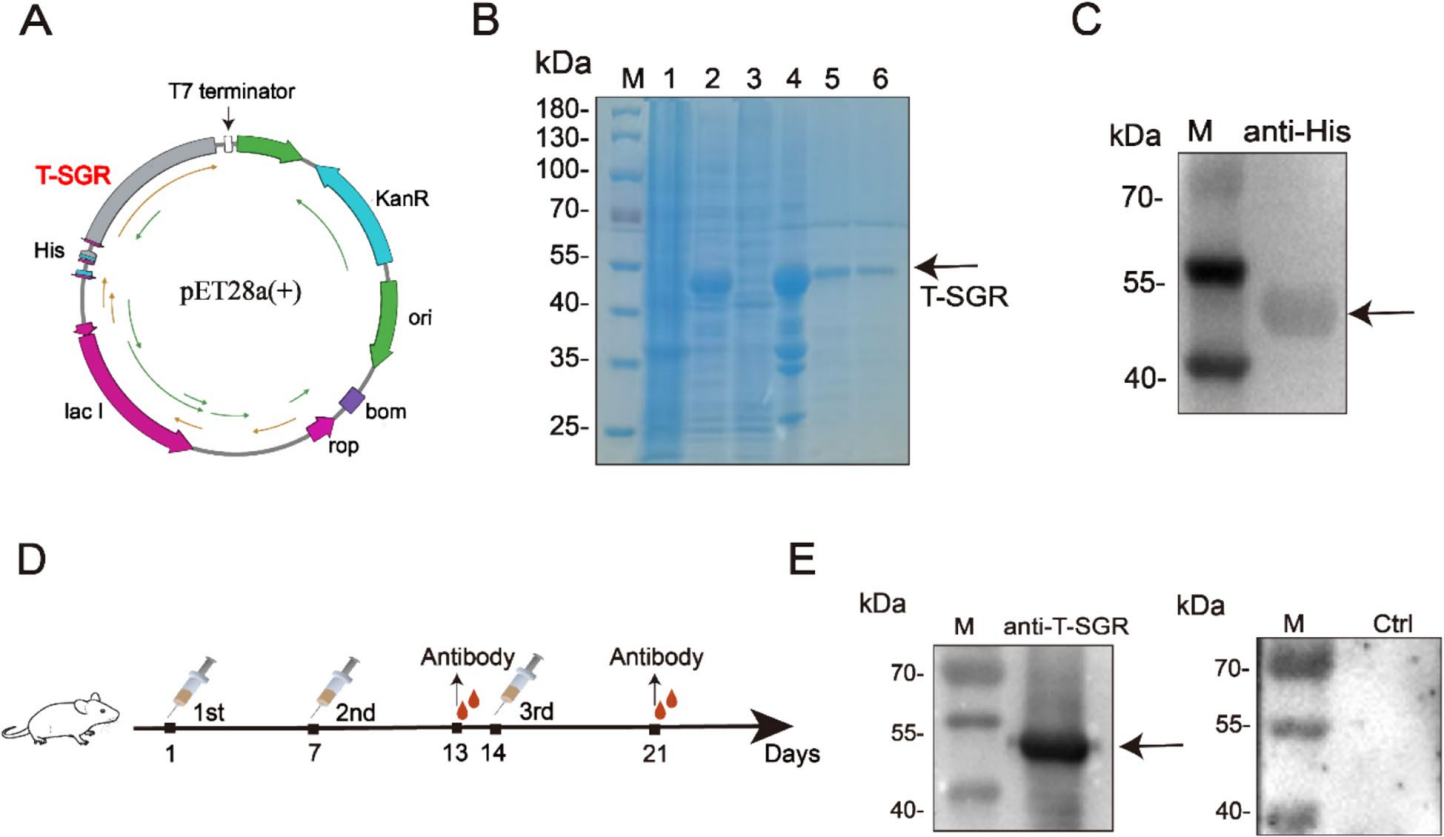
In vitro expression of *T. gondii* T-SGR. **A** Schematic diagram of the pET-28a( +)-T-SGR plasmid construction. **B** SDS–PAGE analysis of the recombinant pET-28a-His-T-SGR protein expressed in *Escherichia coli*. Lane M: protein molecular weight marker (10–150 kDa); lane 1: uninduced bacterial culture; lane 2: bacterial lysate after IPTG induction; lane 3: supernatant after sonication; lane 4: protein inclusion bodies; lane 5 and 6: purified protein samples. **C** Western blot identification of the purified T-SGR–His fusion protein. **D** Flow chart of rat immunization. **E** Western blot analysis of the T-SGR polyclonal antibody

The schematic diagram of the pET-28a-His-T-SGR construct is shown in Fig. 1A. The plasmid was transformed into *E. coli* BL21 cells, and expression was induced with IPTG for 4 h. SDS–PAGE analysis revealed a target band at approximately 45 kDa, and the protein was expressed as inclusion bodies. Purified protein was obtained after Ni–NTA affinity chromatography (Fig. 1B). Western blot analysis showed that the purified T-SGR protein was recognized by an anti-His tag antibody, confirming the successful expression of T-SGR (Fig. 1C). To evaluate the immunogenicity of the recombinant T-SGR protein, Wistar rats were immunized following the protocol shown in Fig. 1D. Western blot was used to detect T-SGR-specific antibodies in rat serum. The results demonstrated that serum from T-SGR-immunized rats specifically recognized the T-SGR protein, with a specific band observed at 45 kDa (Fig. 1E), indicating that anti-T-SGR antibodies were successfully generated.

### *T. gondii* T-SGR mRNA vaccine construction

**Fig. 2:**
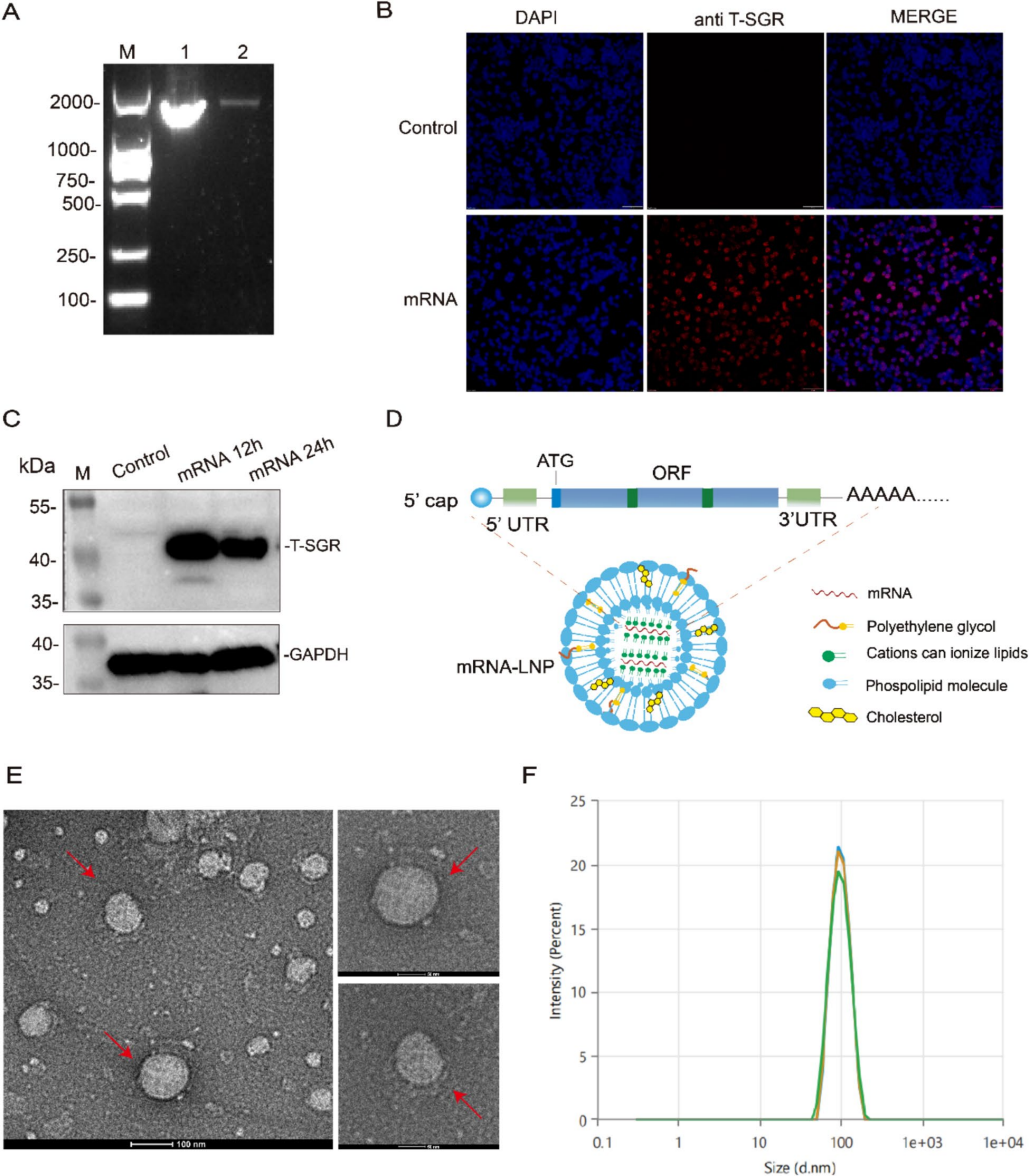
Construction and in vitro expression of the T-SGR mRNA vaccine. **A** In vitro transcription of T-SGR mRNA. Lane M: DNA marker; lane 1: DNA template fragment; lane 2: in vitro transcription product. **B** Analysis of T-SGR mRNA expression in 293 T cells by indirect immunofluorescence assay (IFA). **C** Detection of T-SGR mRNA expression in 293 T cells by western blotting. **D** Schematic diagram of the LNP T-SGR mRNA vaccine construct, which consists of a 5′ cap, untranslated regions (UTRs), open reading frame (ORF), and poly(A) tail. **E** Transmission electron microscopy (TEM) image of LNP-T-SGR mRNA. **F** Particle size analysis of LNP-encapsulated T-SGR mRNA

T-SGR mRNA was prepared using in vitro cotranscriptional capping technology, followed by enzymatic 3′ polyadenylation modification to yield intact, mature T-SGR mRNA. The concentration of T-SGR mRNA was determined to be 1008.9 ng/μL. Denaturing agarose gel electrophoresis confirmed the electrophoretic mobility of the mature mRNA (Fig. 2A). Quality assessment of T-SGR mRNA revealed a structural integrity of 94.4%, 5′ capping efficiency of 94.5%, and a typical bimodal distribution pattern of the poly(A) tail (Supplementary Fig. S2).

To verify whether the constructed mRNA could be expressed in eukaryotic cells, cell transfection assays were performed. Indirect immunofluorescence showed that red fluorescence signals corresponding to the T-SGR protein were observed in transfected HEK 293 T cells at 12 h post-transfection, whereas no such signals were detected in untransfected cells (Fig. 2B). Western blotting revealed a single specific band at approximately 40 kDa in T-SGR mRNA-transfected cells at both 12 h and 24 h (Fig. 2C).

To achieve efficient delivery of mRNA, T-SGR mRNA was encapsulated into lipid nanoparticles (LNPs) via a microfluidic nanoprecipitation system, with the encapsulation schematic shown in Fig. 2D. Transmission electron microscopy (TEM) showed that T-SGR mRNA–LNPs displayed a regular spherical morphology (Fig. 2E). Dynamic light scattering (DLS) demonstrated an average hydrodynamic diameter of 94.33 nm for T-SGR mRNA–LNPs (Fig. 2F), with a polydispersity index (PDI) of 0.052, indicating uniform particle size and favorable dispersity. The encapsulation efficiency of T-SGR mRNA was 92.99%, as measured by RiboGreen fluorescent dye assay, and the total mRNA concentration in T-SGR mRNA–LNPs reached 120.91 μg/mL.

### Detection of IgG antibody levels and antibody subtypes in vaccinated mice

**Fig. 3:**
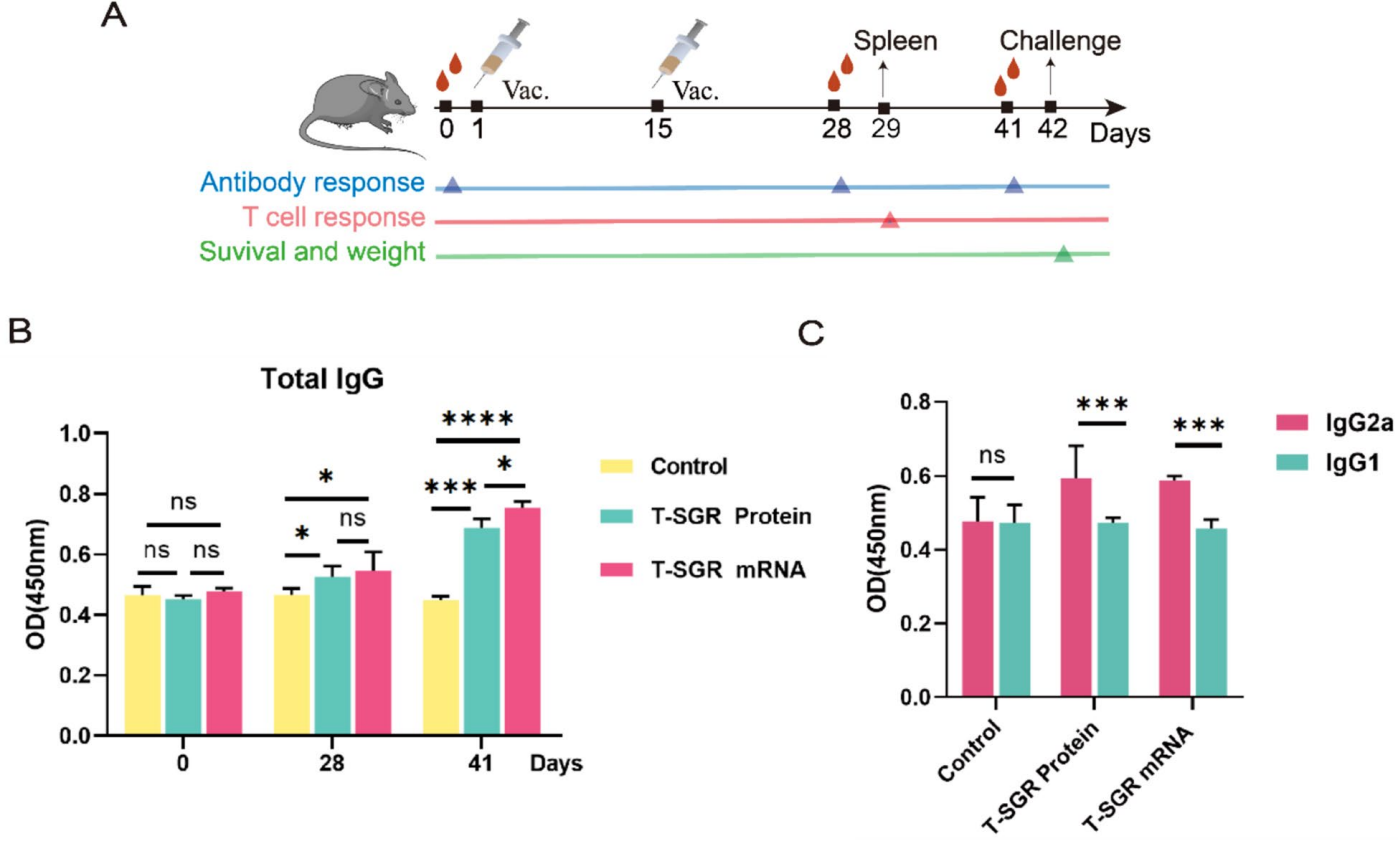
Detection of IgG antibody levels and antibody subtypes in vaccinated mice. **A** Schedule of mice immunization and sample collection. **B** Serum IgG antibody levels measured by ELISA. **C** Serum IgG1 and IgG2a antibody levels detected by ELISA. Data were analyzed by one-way ANOVA followed by Tukey’s multiple comparisons. *P < 0.05, **P < 0.01, ***P < 0.005, ****P < 0.001

Mice immunization and sample collection were performed as shown in Fig. 3A. Serum titers of IgG, IgG1, and IgG2a were determined by ELISA. Both the T-SGR mRNA–LNP vaccine and the recombinant protein vaccine induced specific humoral immune responses in mice 4 weeks after the final immunization, and the antibody titer in the T-SGR mRNA–LNP group was significantly higher than that in the recombinant protein vaccine group (*P* < 0.05) (Fig. 3B). Analysis of IgG subclasses showed that IgG2a levels were significantly higher than IgG1 levels in both vaccine groups (Fig. 3C), indicating that both vaccines tended to induce a Th1-type immune response.

### Cellular immune responses detected in vaccinated mice

**Fig. 4:**
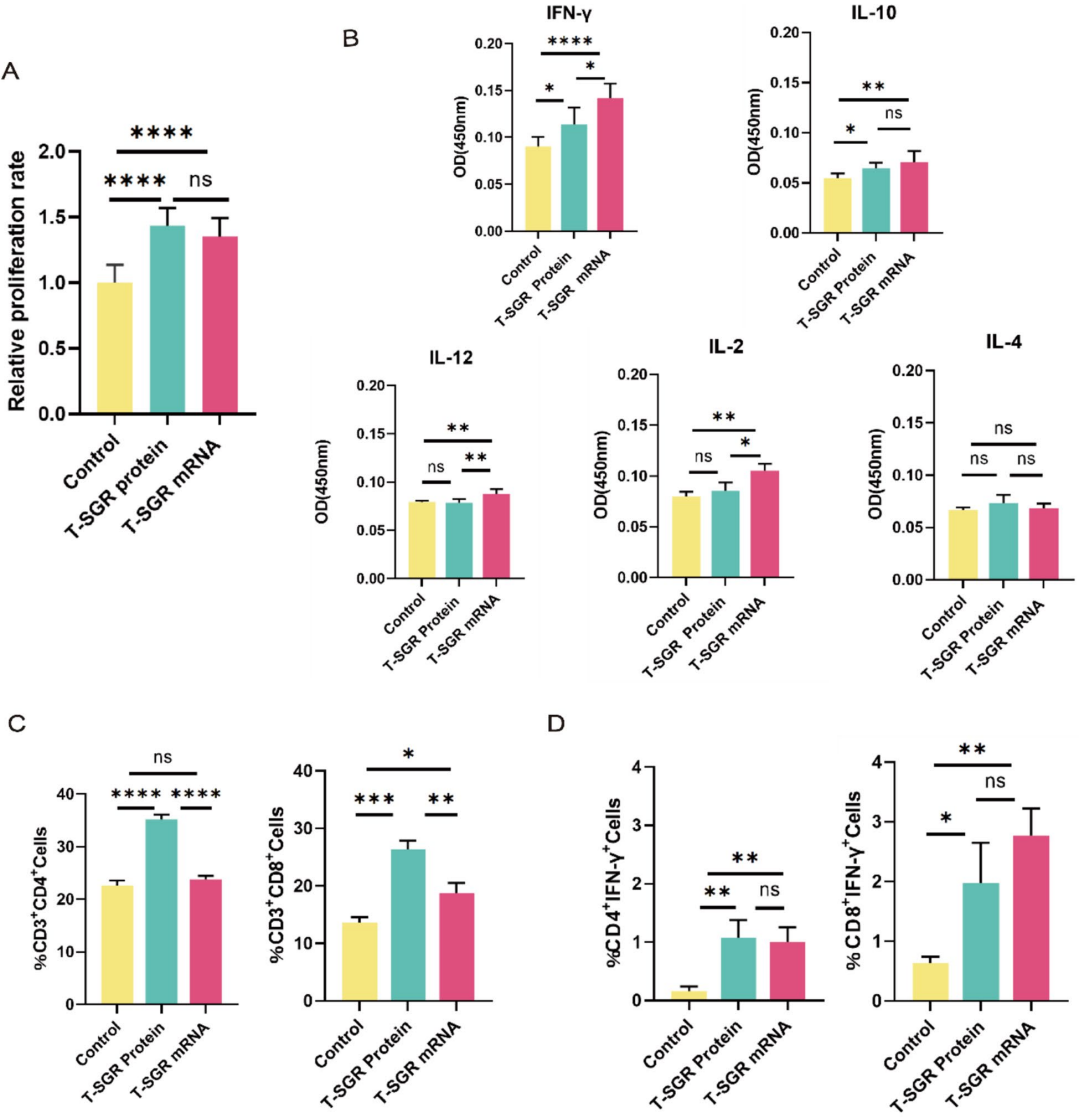
Detection of specific cellular immune responses in vaccinated mice. **A** Splenic lymphocytes were isolated 2 weeks after the final immunization, and antigen-specific lymphocyte proliferation was detected by CCK-8 assay. **B** Cytokine levels (IFN-γ, IL-10, IL-2, IL-12, and IL-4) in culture supernatants following in vitro antigen restimulation. **C** Analysis of T cell subsets in immunized mice by flow cytometry. **D** Analysis of IFN-γ-producing CD4^+^/CD8^+^ T cells in immunized mice by flow cytometry. Data were analyzed by two-way ANOVA. *P < 0.05, **P < 0.01, ***P < 0.005, ****P < 0.001

To evaluate the effect of the T-SGR vaccine on immune cell proliferation, splenic lymphocyte proliferation was assessed using the CCK-8 assay. The results showed that the relative proliferation rates of splenic lymphocytes in both the T-SGR mRNA–LNP vaccine group and the recombinant protein vaccine group were significantly higher than those in the PBS control group (*P* < 0.05), indicating that both vaccines effectively stimulated specific splenic lymphocyte proliferation and laid the foundation for specific immune responses in vivo (Fig. 4A).

To further characterize the regulatory effects of the vaccines on cellular immune responses, cytokine secretion levels in the culture supernatants of splenocytes from immunized mice were measured by ELISA. Compared with the PBS control group, both vaccine groups exhibited significantly elevated secretion of IFN-γ and IL-10. Notably, the T-SGR mRNA–LNP vaccine induced not only higher levels of IFN-γ and IL-10 but also significantly increased secretion of IL-2 and IL-12 compared with the recombinant protein vaccine (Fig. 4B). Cellular immune responses involve not only cytokine secretion but also the activation and differentiation of specific T cell subsets. We therefore performed flow cytometry to analyze antigen-specific T cell subsets in splenocytes from immunized mice. Compared with the PBS control group, the recombinant protein vaccine group showed significant increases in both CD4^+^ and CD8^+^ T cell proportions (*P* < 0.05), whereas the T-SGR mRNA–LNP vaccine group mainly induced a marked elevation in the CD8^+^ T cell proportion (*P* < 0.05) (Fig. 4C). Further analysis of functional T cell responses revealed that both vaccines significantly enhanced IFN-γ secretion by CD4^+^ and CD8^+^ T cells. Moreover, IFN-γ production by CD8^+^ T cells was significantly higher in the T-SGR mRNA–LNP vaccine group than in the recombinant protein vaccine group (*P* < 0.05) (Fig. 4D), demonstrating that the T-SGR mRNA–LNP vaccine is superior in activating cellular immunity and inducing functional T cell responses.

### Protective efficacy of vaccinated mice challenged with *T. gondii*

To evaluate the protective efficacy of the vaccines, mice in each group were intraperitoneally injected with 100 *T. gondii* RH tachyzoites (type I) or 200 ME49 strain (type II,) tachyzoites, and body weight and survival time were recorded daily.

**Fig. 5:**
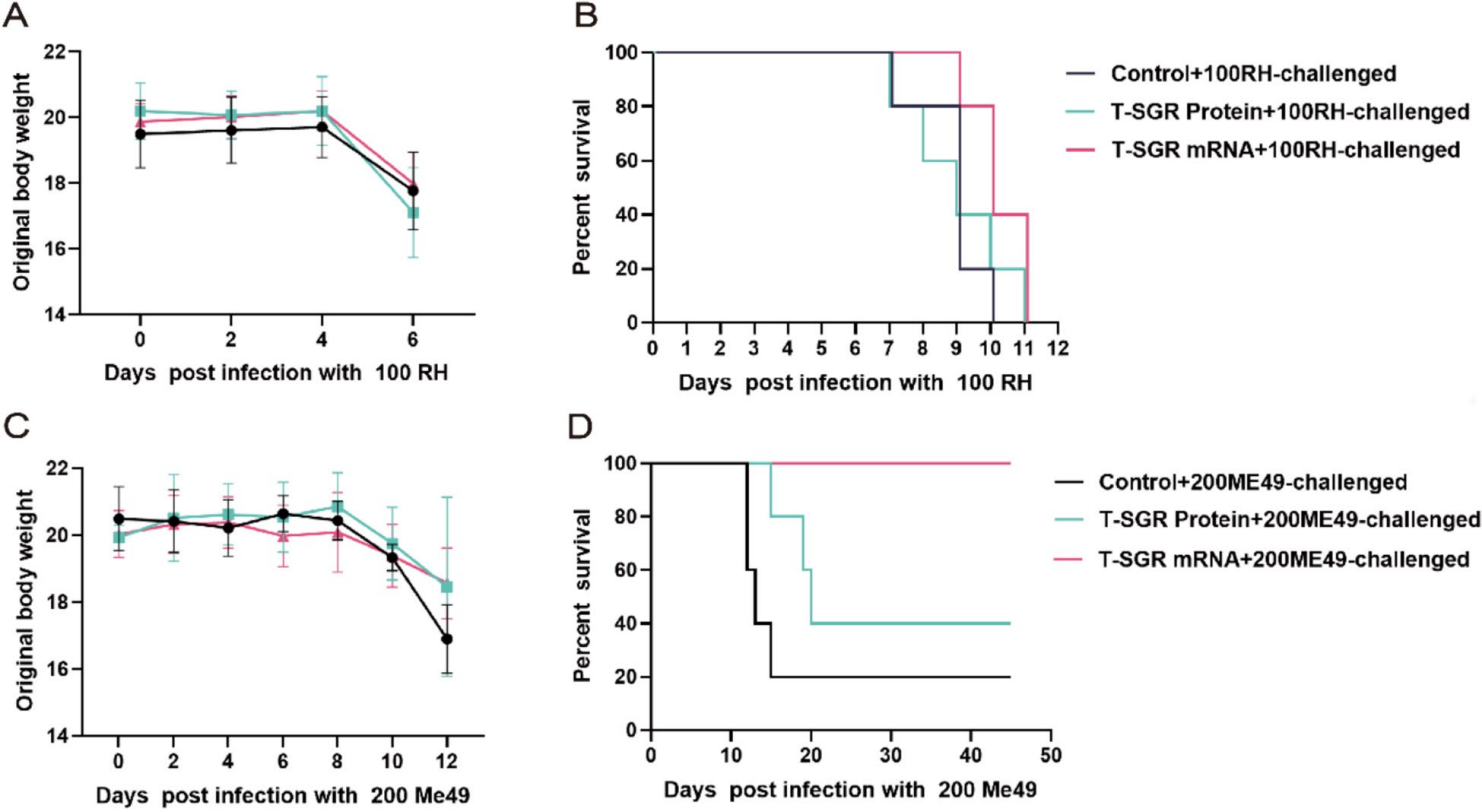
Evaluation of vaccine protective efficacy. **A** Body weight changes in C57BL/6 mice challenged with 100 RH strain tachyzoites 4 weeks after the final immunization. **B** Survival curves of immunized mice challenged with 100 RH strain tachyzoites 4 weeks after the final immunization. **C** Body weight changes in C57BL/6 mice challenged with 200 ME49 strain tachyzoites 4 weeks after the final immunization. **D** Survival curves of immunized mice challenged with 200 ME49 strain tachyzoites 4 weeks after the final immunization. This experiment was performed with five mice per group across two independent replicates. Statistical analyses of survival curves were performed using the Mantel–Cox test

Following challenge with *T. gondii* RH tachyzoites, significant weight loss was observed in all mice on day 6 post-infection. All mice in the PBS control group died within 10 days after challenge, while the T-SGR mRNA–LNP and recombinant protein vaccine groups showed a slight prolongation of survival time compared with the control group (Fig. 5A, B).

Following challenge with *T. gondii* ME49 tachyzoites, severe weight loss was observed in control mice on day 12 post-infection, with a mortality rate of 80%. The survival rate in the T-SGR recombinant protein vaccine group was increased by 20% compared with the control group. Notably, the T-SGR mRNA–LNP vaccine group achieved a 100% survival rate (Fig. 5C, D). Furthermore, brain parasite burden was detected in surviving mice at 45 days after ME49 strain challenge. The results showed that parasite burden was significantly reduced in both the protein vaccine group and the mRNA vaccine group compared with the control group, indicating that both vaccines effectively controlled chronic *T. gondii* infection (Supplementary Fig. S3).

## Discussion

Traditional research on *T. gondii* vaccines has mainly focused on four categories: single-antigen vaccines [25, 26], recombinant protein vaccines [27, 28], DNA vaccines [29, 30], and attenuated live vaccines [31]. All these types of vaccines have obvious limitations and are difficult to meet clinical application requirements. In contrast, multi-epitope vaccines can integrate dominant epitopes from multiple antigens, broaden the scope of immune recognition, and thereby achieve more comprehensive immune protection, providing a new direction for the development of *T. gondii* vaccines [32]. Meanwhile, mRNA vaccines have gradually become a research hotspot owing to their prominent advantages such as endogenous expression and strong immunogenicity.

Accordingly, in this study, we fused the immunodominant fragments of three key *T. gondii* antigens (SAG1, GRA7, and ROP16) to construct the multi-epitope chimeric antigen T-SGR. Using this antigen as the target, we systematically compared the immune effects and protective efficacy of the corresponding mRNA–LNP vaccine and recombinant protein vaccine in a mouse model.

Immune response detection showed that both vaccines induced a strong Th1-biased immune response, as reflected by high IgG2a/IgG1 ratios and elevated IFN-γ secretion [33, 34]. Among them, the mRNA–LNP vaccine induced higher and longer-lasting antibody titers, which was mainly attributed to the differences in delivery and expression patterns between the two vaccines. mRNA vaccines are directly translated in host eukaryotic cells to produce proteins with native-like conformations, which maximally retain immunodominant epitopes and enable efficient antigen processing and presentation, accounting for their stronger immunogenicity. In contrast, as exogenous antigens, recombinant protein vaccines trigger rapid but short-lived humoral immunity and are difficult to induce long-term immune protection.

At the cellular immune level, both vaccines promoted splenic lymphocyte proliferation but differed significantly in activation efficiency. The recombinant protein vaccine mainly activated CD4^+^ T cells via the MHC II pathway, with weak activation of CD8^+^ T cells [35, 36]. In contrast, the mRNA vaccine enabled cross-presentation through the MHC I pathway, efficiently activating CD8^+^ cytotoxic T cells and inducing higher levels of key Th1 cytokines (IFN-γ, IL-12, etc.), thus generating a stronger and more balanced cellular immune response that supports defense against *T. gondii* infection [37, 38].

The results of the in vivo protection assay were highly consistent with the above immune response characteristics, and the mRNA vaccine showed significantly superior protective efficacy compared with the recombinant protein vaccine. In the moderately virulent ME49 strain challenge model, the T-SGR mRNA vaccine achieved 100% mouse survival and significantly reduced brain parasite burden, achieving complete protection. In the highly virulent RH strain challenge model, both vaccines prolonged mouse survival time, and the mRNA vaccine provided better protection; although it did not achieve complete protection, its protective capacity was significantly improved relative to the recombinant protein vaccine.

## Conclusions

The multi-epitope T-SGR antigen represents an ideal candidate for *T. gondii* vaccine development. The T-SGR mRNA–LNP vaccine elicits stronger humoral and cellular immune responses and provides more effective protection than the recombinant protein vaccine. These findings support mRNA–LNP as a promising platform for developing next-generation vaccines against toxoplasmosis.

## Supplementary Information

Additional file1 (PDF 1322 KB)

Additional file2 (DOCX 1400 KB)

## Data Availability

Data supporting the main conclusions of this study are included in the manuscript.

## Abbreviations

CCK-8: Cell Counting Kit-8
DMEM: Dulbecco’s modified Eagle medium
ELISA: Enzyme-linked immunosorbent assay
HEK-293 T cells: Human embryonic kidney 293 T cells
LC–MS: Liquid chromatography–mass spectrometry
LNP: Lipid nanoparticle
PBS: Phosphate-buffered saline
PDI: Polydispersity index
SD: Standard deviation
SDS–PAGE: Sodium dodecyl sulfate—polyacrylamide gel electrophoresis
TEM: Transmission electron microscopy
TMB: 3,3′,5,5′-Tetramethylbenzidine
T-SGR: mRNA vaccine consisting of three antigens of *T. gondii*: SAG1, GRA7, and ROP16
WB: Western blot
