# Supplementary material for: Multi-epitope mRNA vaccine and protein vaccine protect mice against Toxoplasma gondii
Source: Parasit Vectors. 2026 Jun 5;19:329. doi: 10.1186/s13071-026-07447-5 (PMC13459236; doi:10.1186/s13071-026-07447-5)

Control-1

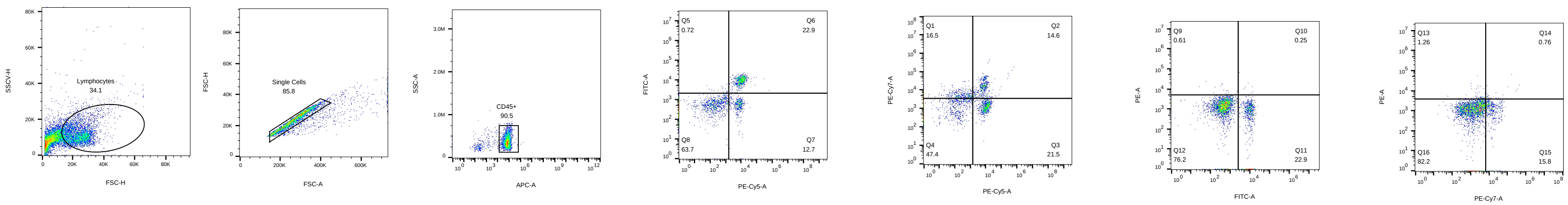

Control-2

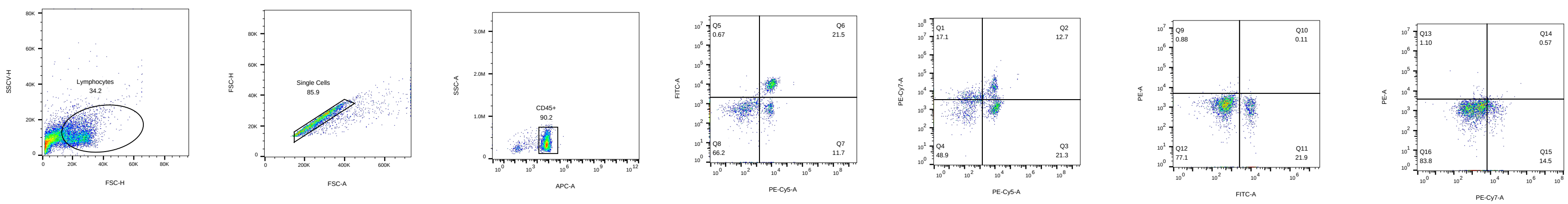

Control-3

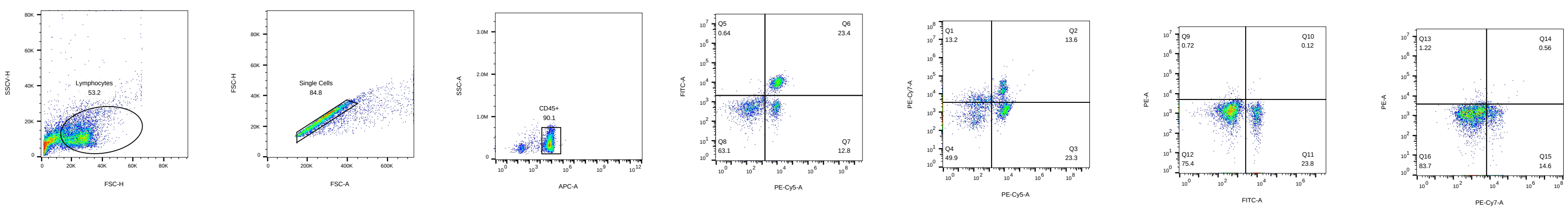

T-SGR Protein-1

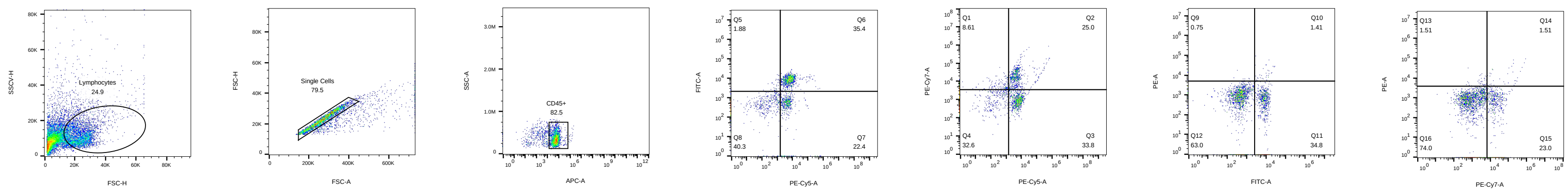

T-SGR Protein-2

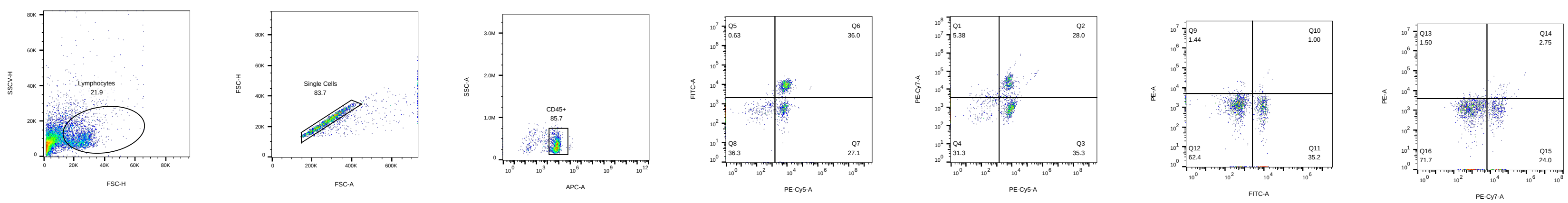

T-SGR Protein-3

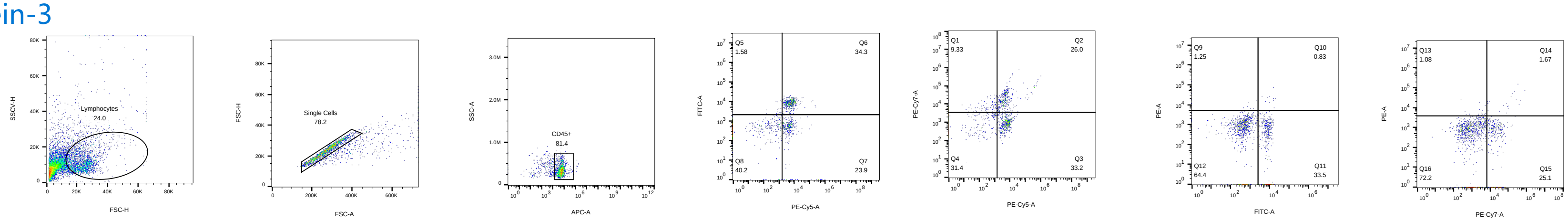

T-SGR mRNA-1

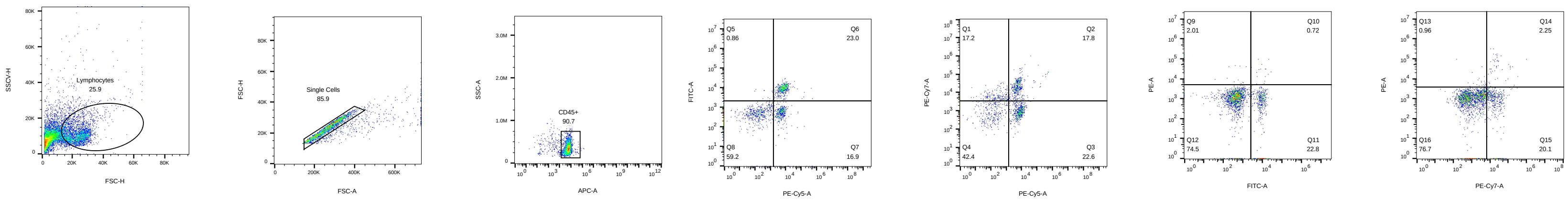

T-SGR mRNA-2

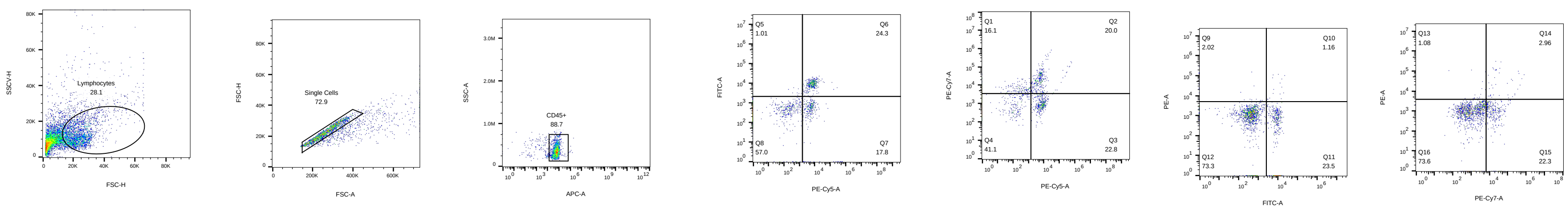

T-SGR mRNA-3

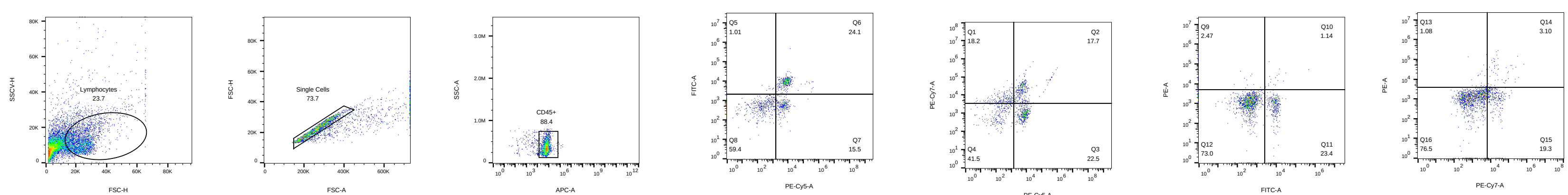

Supplement: Supplementary file 1 [file 13071_2026_7447_MOESM1_ESM.pdf]
