# Supplementary material for: Multi-epitope mRNA vaccine and protein vaccine protect mice against Toxoplasma gondii
Source: Parasit Vectors. 2026 Jun 5;19:329. doi: 10.1186/s13071-026-07447-5 (PMC13459236; doi:10.1186/s13071-026-07447-5)
Supplement: Supplementary file 2 [file 13071_2026_7447_MOESM2_ESM.docx]

IgG

| Concentration(ng/ml) | OD450nm |
| --- | --- |
| 400 | 2.388 |
| 200 | 1.351 |
| 100 | 0.602 |
| 50 | 0.237 |

IgG1

| Concentration(ng/ml) | OD450nm |
| --- | --- |
| 400 | 2.34 |
| 200 | 1.113 |
| 100 | 0.59 |
| 50 | 0.232 |

IFN-γ

OD450nm

| Concentration(ng/ml) | OD450nm |
| --- | --- |
| 400 | 2.529 |
| 200 | 1.369 |
| 100 | 0.452 |
| 50 | 0.148 |

IL-10

| Concentration(ng/ml) | OD450nm |
| --- | --- |
| 400 | 2.511 |
| 200 | 1.353 |
| 100 | 0.46 |
| 50 | 0.155 |

IL-2

| Concentration(ng/ml) | OD450nm |
| --- | --- |
| 400 | 2.61 |
| 200 | 1.38 |
| 100 | 0.447 |
| 50 | 0.142 |

IL-4

| Concentration(ng/ml) | OD450nm |
| --- | --- |
| 400 | 2.555 |
| 200 | 1.371 |
| 100 | 0.45 |
| 50 | 0.146 |

IL-12

| Concentration(ng/ml) | OD450nm |
| --- | --- |
| 400 | 2.53 |
| 200 | 1.371 |
| 100 | 0.459 |
| 50 | 0.172 |

**T-SGR DNA sequence：**

CAAGCCAGAGCCTCATCGGTCGTCAATAATGTCGCAAGGTGCTCCTACGGTGCAGACAGCACTCTTGGTCCTGTCAAGTTGTCTGCGGAAGGACCCACTACAATGACCCTCGTGTGCGGGAAAGATGGAGTCAAAGTTCCTCAAGACAACAATCAGTACTGTTCCGGGACGACGCTGACTGGTTGCAACGAGAAATCGTTCAAAGATATTTTGCCAAAATTAACTGAGAACCCGTGGCAGGGTAACGCTTCGAGTGATAAGGGTGCCACGCTAACGATCAAGAAGGAAGCATTTCCAGCCGAGAGCGCCCCCGGCACCCCCCTGAAGAAGCTCGCGCTGCCGGCTGTTGGTATGGGTGCATCGTATTTTGCCGCTGATAGAATTCTGCCGGAACTAACAGAGCAGCAACAGACAGGCGAAGAACCCCTAACCACCGGCCAGAATGTGAGCACTGTGTTAGGCTTCGCAGCGCTTGCTGCTGCCGCAGCGTTCCTTGGCATGGGTCTCACGAGGACGTACCGACATTTTTCCCCACGCAAAAACAGATCACGGCAGCCTGCACTCGAGCAAGAGGTGCCTGAATCAGGCAAAGATGGGGAGGATGCCCGCCAGAGCGCCCCCGGCACCCCCCAGAGCGCTACATTGGATTCAGGAATACAGTCTCCGGCACAAGAGCGTCGGGGATCCCCTCAAAGACAGATTGCGATGTCGACCGAAAATCCAGCGGATAGCGGCGCCTCGCAGCTTGCCTCCAGTGTTTCTAGTTATGTAGCAGTACAAACTCCTCATGTGAAACGTTCAGAACGCATCCGGCGCGTTCGACTTTCAGAAGAGGGTCTGGAAGAAGTTCAGCAGCTGAAAGCAGCTGCCGCACAGCTTCTCGTAGCGGTTCCGGACTATGAGGCAATGCGGGCTGTTCTGCAAGAGGCGGTCCTCTCAGAACAGAGGGTTGCTACCCGTAAGCGGAAGAGAAAGCAACCTCCAGGAGCGGTGGAGTCAGCTGTTGACGAAGTGTTCCCTCCAAATGAGCGTGTCATGATGATAAATGCCAACGGAGTGCCGATCGCTCTATACAATCGTGGG
